# Retrievable Inferior Vena Cava Filters in Patients with Cancer: Complications and Retrieval Success Rate

**DOI:** 10.1155/2016/6413541

**Published:** 2016-01-21

**Authors:** Ana I. Casanegra, Lisa M. Landrum, Alfonso J. Tafur

**Affiliations:** ^1^Department of Internal Medicine, Cardiovascular Section, Vascular Medicine Program, University of Oklahoma Health Sciences Center, 920 Stanton L. Young Boulevard, WP 3010, Oklahoma City, OK 73104, USA; ^2^Department of Obstetrics and Gynecology, Section of Gynecology Oncology, Stephenson Cancer Center, University of Oklahoma Health Sciences Center, 800 NE 10th Street, Oklahoma City, OK 73104, USA; ^3^Department of Medicine, Vascular Surgery and Medicine Section, NorthShore University HealthSystem, 2650 Ridge Avenue, Evanston, IL 60201, USA

## Abstract

Active cancer (ACa) is strongly associated with venous thromboembolism and bleeding. Retrievable inferior vena cava filters (RIVCF) are frequently placed in these patients when anticoagulation cannot be continued.* Objectives*. To describe the complications and retrieval rate of inferior vena cava filters in patients with ACa.* Methods*. Retrospective review of 251 consecutive patients with RIVCF in a single institution.* Results*. We included 251 patients with RIVCF with a mean age of 58.1 years and a median follow-up of 5.4 months (164 days, IQR: 34–385). Of these patients 32% had ACa. There were no differences in recurrence rate of DVT between patients with ACa and those without ACa (13% versus 17%, *p* = ns). Also, there were no differences in major filter complications (11% ACa versus 7% no ACa, *p* = ns). The filter retrieval was not different between groups (log-rank = 0.16). Retrieval rate at 6 months was 49% in ACa patients versus 64% in patients without ACa (*p* = ns). Filter retrieval was less frequent in ACa patients with metastatic disease (*p* < 0.01) or a nonsurgical indication for filter placement (*p* = 0.04).* Conclusions*. No differences were noted in retrieval rate, recurrent DVT, or filter complications between the two groups. ACa should not preclude the use of RIVCF.

## 1. Introduction

There is a strong association between active cancer (ACa) and venous thromboembolism (VTE) as was historically recognized more than 150 years ago when Armand Trousseau described his eponymous syndrome [1–7]. Cancer-associated thrombosis accounts for about 20% of the entire VTE burden [8]. To date, ACa thromboembolism is a leading cause of death among patients with ACa [9, 10]. The risk of thrombosis as well as the risk of VTE recurrence is increased in this population, driving a high cost in morbidity, hospitalization duration, treatment delay [1, 11]. Paradoxically, ACa not only affects the risk of thrombosis but also increases the likelihood of severe bleeding complications from anticoagulation [12–14]. Patients with cancer associated VTE are often treated with chronic low molecular weight heparin. In the landmark trial by Lee et al. which recruited patients with cancer and acute VTE who were randomized to tinzaparin (449 patients) or warfarin (451 patients) the six-month major bleeding rate was 2.6% and clinically relevant bleeding was 13% [15]. In a single-arm multicenter study with longer follow-up of 334 patients, Francis et al. reported a major bleeding risk of 10% after 214 days median follow-up among patients with cancer receiving prolonged secondary prevention for VTE using a reduced dose of dalteparin (150 IU/kg daily) [16]. The use of inferior vena cava (IVC) filters is often indicated In ACa patients which frequently have both complex thrombotic disease and a major contraindication for anticoagulation, as is currently recommended in the current American College of Chest Physicians Evidenced-Based Clinical Practice Guidelines, 9th Edition, 2012 [17].

Yet, the use of IVC filters is not free of complications. This has reached national attention and retrievable filters are currently recommended over permanent filter with the development of a concrete plan for later removal [18]. In most institutions only a minority of filters are actually removed (8.5–34%) [18, 19] which may lead to an increased rate of filter related complications, including thrombosis at the filter site, erosion into the wall of the vena cava, infection, recurrent lower extremity thrombosis, and migration of the filter, as device related complications increase with dwell time [20]. The objective of our study was to evaluate the rate of IVC filters in patients with and without ACa at a single institution.

## 2. Methods

We included consecutive adult subjects with a retrievable IVC filter placed in our institution from 1 January 2010 to 31 December 2012.

We retrospectively reviewed the electronic medical records (EMR) to document cancer status, comorbidities, indication for the filter placement, complications related to the filter, thrombotic events while the filter was in place, retrieval of the filter, anticoagulation, and date of death as documented in EMR or in the Social Security Death Index (SSDI). We reviewed all the available imaging studies related to VTE and filter complications including the baseline venogram to assess complications at insertion time.

Since 2010, we have an established filter clinic in our institution. All the patients with a RIVCF have a 3-month follow-up with a Vascular Medicine specialist if the filter is still in place to determine whether the filter needs to stay permanently or to plan for retrieval after evaluating risks and benefits. This decision is documented in the EMR.

Active cancer was defined as metastatic disease or any cancer treatment within 6 months before the filter placement, excluding nonmelanoma cancers of the skin [21]. In the subgroup of patients with ACa we obtained additional information including the type of cancer, stage, grade, and treatment, and we calculated the Khorana and Ottawa scores for stratification of cancer specific thrombosis likelihood [22, 23]. Khorana score considers the site of the cancer, platelet count, hemoglobin, leukocyte count, and BMI and divides patients in risk categories (low, moderate, high, and very high). Ottawa score takes into account site of the tumor, stage, and prior VTE to stratify the patients in high or low recurrence rate for VTE.

Our primary outcome was major filter complications characterized by tilting or thrombosis preventing retrieval, migration, embolization, fracture, and penetration of the cava wall. Secondary outcomes were filter retrieval, a documented decision to leave it in place permanently, incident VTE, and a combined endpoint of incident VTE or filter complication. Incident thromboembolic events (DVT or PE) were defined as new events confirmed by an imaging study and involved a previously unaffected segment. All outcomes were deemed present by mutual agreement between the authors. Patients were followed until they died or until the filter was removed or until the closure of the study on 1 July 2013. Retrieval rate was calculated in surviving patients.

Filter complications were defined as follows: penetration of the strouts >3 mm through the IVC wall, tilting of more than 15 degrees, migration of the filter of over 2 cm from initial location, embolization to a different location (heart and lung), and thrombosis identified by imaging studies.

Statistical analysis was performed with SAS (version 9.3, SAS Institute, Cary, NC) and JMP (version 11, SAS Institute, Cary, NC). A *p* value < 0.05 was considered statistically significant. Quantitative variables are expressed as mean ± standard deviation, nonparametric variables are reported as median and interquartile range (IQR), and qualitative variables are presented as percentages. Univariate analyses of continuous variables were conducted with Student's *t*-tests to compare means and Wilcoxon's test was used for nonparametric variables. Categorical variables were analyzed with *χ* ^2^ or Fisher's exact tests. Time-to-event analysis was performed with the Kaplan-Meier method for time to filter retrieval accounting for death as competing event. The Kaplan-Meier curves were evaluated with a log-rank *χ* ^2^ test.

An exploratory stepwise multivariate analysis with logistic regression was performed to identify factors independently associated with major filter complications. Hosmer-Lemeshow test was used to assess the model's goodness of fit. The variables explored were those with a *p* < 0.3 in the univariate analysis; they were retained in the model if *p* < 0.35.

## 3. Results

### 3.1. Cohort and Patients with Cancer Description

We included 267 patients who received retrievable IVC filters (RIVCF). Five percent of the filters (*n* = 16) were placed prophylactically and were excluded. Most of these excluded patients were patients with trauma (*n* = 13). The mean age was 58.1 ± 16.3 years, and the median follow-up was 5.4 months (164 days, IQR: 34–385). A third of the patients (36%) died during follow-up. There were 121 males (48.2%), 222 (88.5%) had a DVT, and 91 (36.3%) had a PE at baseline. One-third of the patients (*n* = 87, 34.7%) had ACa (Table 1). Patients with ACa were older (61.8 ± 13.5 versus 56.1 ± 17.4 years, *p* < 0.01), were more frequently females (67.8% versus 43.3%, *p* < 0.01), and more likely to have PE at baseline (57.5% versus 25%, *p* < 0.01). One-third of the patients with ACa (*n* = 28, 32.2%) were on chemotherapy at the time of the filter placement. The primary sites were gynecologic (*n* = 36, 41%), central nervous system (*n* = 11, 13%), gastrointestinal tract and pancreas (*n* = 10, 12%), urological (*n* = 6, 7%), lung (*n* = 6, 7%), and other sites (*n* = 18, 21%). Half of these patients had metastatic disease (*n* = 44, 51%). The RIVCF more commonly used in our institution were eclipse (*n* = 143, 58%), Optease (*n* = 45, 18%), Celect (*n* = 29, 12%), and G2 (*n* = 23, 9%). There was no filter preference based on ACa status.

Indications for filter placement are in Table 1. Active bleeding was the most common indication in patients without cancer (53% versus 39%, *p* = 0.035), and high bleeding risk was more common in patients with ACa (20% versus 9%, *p* < 0.01). Patients with ACa were more likely to receive anticoagulation after the event (70% versus 54%, *p* = 0.01) than patients without ACa.

More patients with ACa died during follow-up (55% versus 26%, *p* < 0.01) as demonstrated in Table 2. There was no difference in age, gender, BMI, DVT, history of bleeding, cancer type, chemotherapy, anticoagulation, type of filter, complications, or VTE recurrence between the patients who died and those who survived. Patients with metastatic disease were more likely to have bilateral DVT at presentation (70% versus 29%, *p* = 0.022).

### 3.2. Retrieval Rates

There was no difference in filter retrieval between groups in the Kaplan-Meier analysis (log-rank: 0.16). The retrieval rate at 6 months was 49% versus 64% (*p* = ns) in patients with and without ACa. The time elapsed to filter retrieval (median: 33 days [IQR: 14–63] versus 38 days [IQR: 18–93], *p* = ns) was not different.

The success rate for the first retrieval attempt was 96%. Of the 10 retrieval failures, 5 filters were left permanently and 5 filters were successfully retrieved in a second attempt after 1 to 7 months of anticoagulation.

More patients with ACa died with the filter in place but the difference was not statistically significant (43% versus 21%, *p* = ns). In patients with ACa, filter retrieval was less frequent if they had metastatic disease (OR, *p* = 0.04) or a nonsurgical indication for filter placement (OR, *p* = 0.02). There was no difference in retrieval rate by filter type. Patients without removal of the filter had lower platelets (*p* = 0.011) and were more likely to have metastatic cancer (*p* = 0.038) as described in Table 3. These two variables remained significantly associated with retrieval of the filter in an exploratory multivariate analysis

### 3.3. VTE Recurrence

There was no difference in the Kaplan-Meier analysis for new VTE events between patients with ACa and those without (log-rank: 0.56). More patients with ACa were diagnosed with a new PE (5% versus 0.6%, *p* = 0.05); DVT recurrences were not statistically different (13% versus 17%, *p* = ns).

The patients with ACa and a new VTE were more likely to have a PE at baseline (OR, *p* = 0.02) and to have a decision made of leaving the filter in place (69% versus 30%, *p* < 0.01). Filter complications were more common in this group.

### 3.4. IVC Filter Complications

There was no difference in major filter complications between patients with ACa and those without ACa (17% versus 18%, *p* = ns), as depicted in Table 2. Penetration of the filter through the IVC wall was more commonly found in patients with ACa. The time to complication was not different in patients with and without ACa (*p* = 0.82).

The patients with ACa and filter complications were not different from those without filter complications except from having a higher prevalence of intermediate and high Khorana score (Table 4). There were no differences in type of filter or primary site. More patients with filter complications also had a new VTE (53% versus 8%, *p* < 0.01) (Table 4).

In an exploratory multivariate analysis performed, the best-fitting model to predict filter complications in patients with active cancer included the presence of a new DVT, use of statins, and a medium or high Khorana score.

## 4. Discussion

The main finding in our cohort is that ACa did not affect the incidence of filter related complications or the retrieval rate. Those patients with filter related complications were less likely to have a successful filter retrieval and more likely to develop an incident VTE event. Overall filter retrieval rate in our institution was 73% at one year and ACa was not a predictor of retrieval failure. Among patients with ACa, baseline platelet level, nonsurgical indications for filter placement, and metastatic disease were strong predictors of a lower likelihood of retrieval.

The rate of complications was 17.6% in the overall population, which was within the 14 to 50% range described in the literature. It is possible that our complication rate is in the lower end because the dwelling time was short, and complications are associated with prolonged dwell time [24]. Retrospective reports of filter associated complications have had limited follow-up, 1 to 134 days in a systematic review including 284 filters [25], and there is no consensus on the definition of complication [25, 26] nor mandatory report [20]. As with the study published by Abtahian et al., we did not find differences in the complication rates between patients with and without ACa [27].

The rate of filter-associated complications is usually considered to be a time dependent event. In a FDA Manufacturer and User Facility Device Experience (MAUDE) evaluation of 842 filter-associated complications, only 7% occurred within the first 30 days. In our database the time to complication was similar between the patients with and without cancer and 45% were after the first 30 days. There is paucity of data reviewing the rate of filter-associated complications among patients with cancer. In a retrospective study, which included 308 patients with cancer who received an IVC filter, there were 22 (7.1%) complications including 14 cases of IVC thrombosis [28]. The follow-up time, however, was shorter than ours, which limits adequate comparison. In a smaller, retrospective cancer specific study including 55 patients with stage III or IV cancer who required IVC filter placement, the rate of thrombotic complications was also 7% but with a more restrictive definition [29]. Neither of these studies clarified the retrieval rate. Abtahian et al. followed their patients for a longer time and found a rate of complications that was similar to our study. As discussed, the complication rate seems to be a function of timely removal. The implementation of our filter clinic has generated a timely and more aggressive retrieval strategy. It is plausible that the local strategy justifies our low rate of filter related complications.

In our study, the retrieval rate was lower in patients with metastatic disease. These patients often have a poor prognosis and hence have a shorter survival after filter placement which may have prevented retrieval. This finding was also noted in the retrospective study by Abtahian et al., where retrieval attempts were lower in patients with metastatic disease versus those with limited disease (21% versus 36%, *p* < 0.001) [27].

Our general retrieval rate was consistent with other medical centers with IVC filter protocols for follow-up [30–32] and higher than national averages. In a systematic review of 6834 RIVCF in 37 studies, the mean retrieval rate was only 34% [33]. We found no difference in the retrieval rate between patients with and without ACa. Abtahian et al., in their retrospective study comparing patients with and without ACa, found a lower retrieval rate in patients with ACa [27]. This may reflect local practice, a higher proportion of patients with metastatic disease, or different indications for filter placement. In our study a higher proportion of filters were placed perioperatively for anticoagulation interruption, and those patients had a higher retrieval rate than patients with bleeding or bleeding risk.

The most common indication for filter placement in our study was bleeding in both patients with and those without cancer. In a retrospective study on 103 patients with gynecological malignancies who required an IVC filter, the most common reason for placement was contraindication to anticoagulation due to hemorrhage (44%) [34]. Indeed, the high likelihood of bleeding among patients with cancer who require anticoagulation is well recognized [14]. In a study by Prandoni et al. [12] major bleeding was twice as common in patients with cancer compared to patients without cancer (15.7/100 versus 8.6/100 patients/year), with a hazard ratio for major bleeding of 4.8 (95% CI: 2.3–10.1) in patients with extensive cancer. In our study, patients with thrombocytopenia at baseline or patients with nonsurgical indications for filter placement were less likely to have the filter retrieved. This may indicate that the patient was at risk for persistent bleeding and thus the filter may still be indicated. More patients who were started on anticoagulation within the first month after the event had the filter retrieved. This suggests the patient had a reduced risk of bleeding and the initial indication for the filter was no longer present.

One of the strengths of our study is that all records were available for review. Furthermore, most of the patients have their follow-up in the institution, as well as a three-month follow-up if the filter was still in place as part of our quality improvement project. As the study was retrospective, the usual practice by the different physicians was not modified. Also it is a single center study and thus may reflect local practices. Four different filters were used and that may introduce heterogeneity, but there was no preference for a type of filters by ACa status. Because this is a retrospective study, there is a risk for selection bias. We may have overdiagnosed the number of complications including complications that were not clinically significant by reviewing all the images available from the time of the filter placement. Despite this, our complication rate was similar to what is reported in the literature and not different between patients with and without cancer. Because our institution is a level 1 trauma center, many trauma patients are referred from different regions of the state. Some of these patients were lost to follow-up or follow-up information was incomplete. As most of the indications for IVC filters in trauma patients are short lived, usually their filters are removed before discharge.

## 5. Conclusion

In patients with ACa IVCF placement is an acceptable intervention, as the complications and overall retrieval rate do not differ significantly from the patients without cancer. Predictors of low retrieval rate such as metastatic disease, recurrent VTE, and anemia at baseline should be considered at the time of filter placement to guide the judicious use of the IVCF.

## Figures and Tables

**Table 1:** Clinical characteristics of the subjects by cancer status.

	Active cancer (*n* = 87)	No cancer (*n* = 164)	*p* value
Follow-up (median, IQR)	203 (31–397)	156 (34–349)	0.52
Age, years (mean, SD)	62 (14)	56 (17)	<0.01
Male gender	28 (32)	93 (57)	<0.01
VTE event	87 (96)	164 (93)	0.22
DVT	74 (85)	148 (90)	0.57
Bilateral	24 (32)	41 (28)	0.76
IVC	3 (2)	2 (1)	0.34^*∗*^
Proximal	60 (82)	127 (87)	0.28
PE	50 (57)	41 (25)	<0.01
Filter indication			
Surgery	34 (39)	62 (38)	0.84
Bleeding	34 (39)	87 (53)	0.035
Bleeding risk	17 (20)	13 (8)	<0.01
Failed anticoagulation	2 (2)	2 (1)	0.51^*∗*^
Other^a^	2 (2)	7 (4)	0.42
Bleeding + bleeding risk	51 (59)	100 (61)	0.71
Comorbidities			
COPD	6 (7)	12 (7)	0.90
CHF	7 (8)	20 (12)	0.31
CAD	4 (5)	30 (18)	<0.01^*∗*^
CKD	9 (10)	26 (16)	0.23
Liver disease	1 (1)	9 (5)	0.17^*∗*^
Anticoagulation	61 (70)	88 (54)	0.01
*Cancer related variables*			
Chemotherapy	28 (32)		
Metastatic	44 (51)		
Khorana score			
Low (0)	9 (10)		
Intermediate (1-2)	60 (69)		
High (≥3)	17 (20)		
Ottawa score ≥1	50 (57)		

*Note.* Values are *n* (%) unless otherwise specified. IQR: interquartile range; SD: standard deviation; *∗*: Fisher's exact test, a: other indications included poor cardiopulmonary reserve, massive PE, and thrombectomy.

**Table 2:** Outcomes by active cancer status.

	Active cancer (*n* = 87)	No cancer (*n* = 164)	*p* value
Death	48 (55)	42 (26)	<0.01
Time to death, days (median, IQR)	78 (24–308)	42 (8–86)	<0.01
Died with filter in place	37 (43)	35 (21)	0.45
Filter status changed to permanent	13 (15)	13 (8)	0.59
Filter retrieved	33 (38)	94 (57)	<0.01
Filter retrieved or changed to permanent	46 (53)	107 (65)	0.056
Time to filter retrieval, days (median, IQR)	33 (14–63)	38 (18–93)	0.16
Time to retrieval or changed to permanent, days (median, IQR)	45 (28–94)	92 (39–226)	0.03
*Complications*			
Incident VTE	14 (16)	29 (18)	0.75
DVT	11 (13)	28 (17)	0.35
PE	4 (5)	1 (0.6)	0.05^*∗*^
*N* patients with filter complications	15 (17)	30 (18)	0.83
Filter complications			
Migration	1 (1)	2 (1)	1^*∗*^
Embolization	1 (1)	1 (0.6)	1^*∗*^
Fracture	0	2 (1)	0.55^*∗*^
Thrombosis	9 (10)	15 (9)	0.83^*∗*^
Tilting	2 (2)	12 (7)	0.10^*∗*^
Penetration	4 (4)	1 (0.6)	0.05^*∗*^
Other	1 (1)	3 (2)	1^*∗*^
Major filter complications	10 (11)	12 (7)	0.26
*N* patients with complications or incident VTE	19 (21)	43 (26)	0.44

Note: values are *n* (%) unless otherwise specified. IQR: interquartile range; *∗*: Fisher's exact test.

**Table 3:** Clinical characteristics in patients with active cancer (*n* = 87) with and without filter complications.

	With filter complications (*n* = 15)	Without filter complications (*n* = 72)	*p* value
Age, years (mean, SD)	60 (15)	62 (13)	0.65
Male gender	7 (47)	21 (30)	0.19
BMI (median, IQR)	30 (27–34)	30 (25–35)	0.88
VTE event			
DVT	11 (73)	63 (88)	0.22^*∗*^
Bilateral	3 (27)	21 (33)	1^*∗*^
IVC	0	3	1^*∗*^
Proximal	10 (91)	50 (81)	0.68^*∗*^
PE	9 (60)	41 (56)	0.82
Filter indication			
Surgery	4 (27)	30 (42)	0.39^*∗*^
Bleeding	8 (53)	26 (36)	0.21
Bleeding risk	2 (13)	15 (21)	0.73^*∗*^
Failed anticoagulation	1 (7)	1 (1)	0.32^*∗*^
Other	1 (7)	1 (1)	0.32^*∗*^
Medications			
Antiplatelet	3 (21)	9 (12)	0.40^*∗*^
Statins	5 (36)	11 (15)	0.072
Laboratory			
WBC mean (median, IQR)	8.5 (6.8–10.8)	8.1 (5.6–12.4)	0.86
Hemoglobin mean (mean, SD)	10.3 (2.5)	10.1 (1.7)	0.77
Platelets mean (median, IQR)	237 (181–311)	217 (140–325)	0.95
Creatinine mean (median, IQR)	0.83 (0.07–1.1)	0.9 (0.7–1.2)	0.85
Bilirubin (median, IQR)	0.6 (0.03–0.8)	0.6 (0.4–1.1)	0.20
Chemotherapy	5 (33)	23 (31)	0.92
Metastatic	6 (40)	38 (53)	0.37
Khorana score, intermediate and high	10 (71)	67 (93)	0.035^*∗*^
Ottawa score ≥1	8 (53)	42 (58)	0.72
Follow-up time (median, IQR)	203 (98–357)	200 (28–443)	0.84
Anticoagulation within 1 month	12 (80)	49 (68)	0.36
*Outcomes*			
Death	5 (33)	43 (60)	0.062
Time to death (median, IQR)	203 (164–229)	67 (24–311)	0.23
Died with filter in place	2 (13)	35 (49)	0.07^*∗*^
Filter status changed to permanent	4 (27)	9 (13)	1^*∗*^
Filter retrieved	7 (47)	26 (36)	0.44
Time to filter retrieval, days (median, IQR)	33 (21–60)	31 (9–70)	0.74
Time to retrieval or changed to permanent, days (median, IQR)	55 (31–72)	45 (25–108)	0.93
*Complications*			
Incident VTE	8 (53)	6 (8)	<0.01
DVT	7 (47)	4 (6)	<0.01
PE	1 (6.7)	3 (4)	0.52

Note: values are *n* (%) unless otherwise specified. IQR: interquartile range; SD: standard deviation; *∗*: Fisher's exact test.

**Table 4:** Clinical characteristics in patients with active cancer (*n* = 87) with and without filter retrieval.

	Filter retrieved (*n* = 33)	Filter in place (*n* = 54)	*p* value
Age years, mean (SD)	61 (12)	62 (15)	0.63
Male gender	7 (21)	21 (39)	0.086
BMI mean (median, IQR)	30 (25–35)	30 (26–34)	0.89
VTE event			
DVT	27 (81)	47 (87)	0.51
Bilateral	8 (30)	16 (34)	0.67
IVC	0	3 (6)	1^*∗*^
Proximal	21 (77)	39 (85)	0.38
PE	20 (60)	30 (56)	0.64
Filter indication			
Surgery	18 (55)	16 (30)	0.021
Bleeding	12 (36)	22 (41)	0.68
Bleeding risk	4 (12)	13 (24)	0.27^*∗*^
Failed anticoagulation	1 (3)	1 (2)	1^*∗*^
Other	0	2 (4)	0.52^*∗*^
Medications			
Antiplatelet	3 (9)	9 (17)	0.36^*∗*^
Statins	6 (18)	10 (19)	0.93
Laboratory			
WBC mean (median, IQR)	7.8 (5.6–10.4)	9.1 (5.7–13.2)	0.17
Hemoglobin mean (median, SD)	10.0 (2.2)	10.1 (1.6)	0.91
Platelets (median, IQR)	271 (188–418)	198 (129–279)	0.011
Creatinine (median, IQR)	0.76 (0.61–1.0)	0.9 (0.7–1.2)	0.024
Bilirubin (median, IQR)	0.4 (0.3–0.7)	0.7 (0.5–1.1)	<0.01
Chemotherapy	7 (22)	21 (39)	0.087
Metastatic	12 (36)	32 (59)	0.038
Khorana score, intermediate and high	29 (88)	48 (91)	0.72^*∗*^
Ottawa score ≥1	16 (49)	34 (63)	0.18
Follow-up time (median, IQR)	363 (203–537)	62 (24–305)	<0.01
Anticoagulation within 1 month	30 (91)	31 (57)	<0.01
*Outcomes*			
Death	11 (33)	37 (69)	<0.01
Time to death (median, IQR)	274 (88–412)	34 (24–243)	0.14
*Complications*			
Incident VTE	3 (9)	11 (20)	0.23^*∗*^
DVT	3 (9)	8 (15)	0.52^*∗*^
PE	0	4 (7)	0.29^*∗*^
*N* patients with filter complications	7 (21)	8 (15)	0.44
Filter complications			
Migration	1 (3)	0	0.40^*∗*^
Embolization	1 (3)	0	0.40^*∗*^
Fracture	0	0	
Thrombosis	1 (3)	7 (13)	0.31^*∗*^
Tilting	1 (3)	0	0.15^*∗*^
Penetration	3 (8)	1 (2)	0.30^*∗*^
Other	1 (3)	0	0.40^*∗*^
Major filter complications	3 (9)	7 (13)	0.73^*∗*^
*N* patients with complications or incident VTE	8 (24)	11 (20)	0.67

Note: values are *n* (%) unless otherwise specified. IQR: interquartile range; *∗*: Fisher's exact test.
